# Molecular phylogeny and timing of diversification in Alpine *Rhithrogena* (Ephemeroptera: Heptageniidae)

**DOI:** 10.1186/s12862-016-0758-1

**Published:** 2016-09-21

**Authors:** Laurent Vuataz, Sereina Rutschmann, Michael T. Monaghan, Michel Sartori

**Affiliations:** 1 Musée cantonal de zoologie, Palais de Rumine, Place de la Riponne 6, 1014 Lausanne, Switzerland; 2 Department of Ecology and Evolution, Biophore, University of Lausanne, 1015 Lausanne, Switzerland; 3 Leibniz-Institute of Freshwater Ecology and Inland Fisheries (IGB), 12587 Berlin, Germany; 4 Berlin Center for Genomics in Biodiversity Research, 14195 Berlin, Germany; 5 Department of Biochemistry, Genetics and Immunology, University of Vigo, 36310 Vigo, Spain

**Keywords:** Dated phylogeny, Speciation, Diversification, Lineage-through-time, Pleistocene glaciations, Freshwater insects, Headwater, Mayfly, Ephemeroptera

## Abstract

**Background:**

Larvae of the Holarctic mayfly genus *Rhithrogena* Eaton, 1881 (Ephemeroptera, Heptageniidae) are a diverse and abundant member of stream and river communities and are routinely used as bio-indicators of water quality. *Rhithrogena* is well diversified in the European Alps, with a number of locally endemic species, and several cryptic species have been recently detected. While several informal species groups are morphologically well defined, a lack of reliable characters for species identification considerably hampers their study. Their relationships, origin, timing of speciation and mechanisms promoting their diversification in the Alps are unknown.

**Results:**

Here we present a species-level phylogeny of *Rhithrogena* in Europe using two mitochondrial and three nuclear gene regions. To improve sampling in a genus with many cryptic species, individuals were selected for analysis according to a recent DNA-based taxonomy rather than traditional nomenclature. A coalescent-based species tree and a reconstruction based on a supermatrix approach supported five of the species groups as monophyletic. A molecular clock, mapped on the most resolved phylogeny and calibrated using published mitochondrial evolution rates for insects, suggested an origin of Alpine *Rhithrogena* in the Oligocene/Miocene boundary. A diversification analysis that included simulation of missing species indicated a constant speciation rate over time, rather than any pronounced periods of rapid speciation. Ancestral state reconstructions provided evidence for downstream diversification in at least two species groups.

**Conclusions:**

Our species-level analyses of five gene regions provide clearer definitions of species groups within European *Rhithrogena*. A constant speciation rate over time suggests that the paleoclimatic fluctuations, including the Pleistocene glaciations, did not significantly influence the tempo of diversification of Alpine species. A downstream diversification trend in the hybrida and alpestris species groups supports a previously proposed headwater origin hypothesis for aquatic insects.

**Electronic supplementary material:**

The online version of this article (doi:10.1186/s12862-016-0758-1) contains supplementary material, which is available to authorized users.

## Background

Mountainous areas are hotspots of biodiversity and endemism (e.g. [1, 2]), and headwater streams are increasingly recognized for their contribution to freshwater biodiversity [3–5]. In Europe, the Alps have strongly influenced the evolution of many taxa, acting as a barrier or refuge for populations during climatic fluctuations [6, 7]. Pleistocene glaciations are recognized as important promoters of population divergence and ultimately speciation through repeated isolation in allopatric refugia, increasing speciation rates in a variety of organisms (e.g. [6, 8–11]). However, the role of recent glaciations in speciation has also been largely debated [12–17], and a number of studies favor a model of constant diversification rate during the Quaternary ice ages (e.g. [18–22]).

The processes that drive speciation in aquatic insects remain poorly understood, despite the significant contribution of aquatic insect﻿s to global insect diversity [23]. Several studies of aquatic insects have uncovered evidence for the important influence of glaciations on divergence patterns at the level of both populations (e.g. [24–27]) and species (e.g. [28–30]). In mayflies (Ephemeroptera), population divergence within particular species has often been interpreted to result from their separation into distinct refugia during the Pleistocene (e.g. [31, 32]). Although some authors have suggested that Pleistocene glaciations promoted mayfly speciation in the Palearctic [33] and Australasia [34], extensive analyses of speciation rates during the Quaternary are lacking. Earlier workers proposed that the ancestral position of cold-adapted aquatic insect lineages was in headwaters, with subsequent downstream diversification into warmer waters (e.g. [35–37]). This headwater origin hypothesis has been supported by more recent studies of European caddisflies (Trichoptera, Hydropsychidae) [38, 39], and in some, but not all, lineages of tropical diving beetles (Coleoptera, Dytiscidae) in New Guinea [40]. In contrast, there was no support for the hypothesis in a study of tropical heptageniid mayflies (Heptageniidae) in Madagascar [41]. Findings to date suggest that the headwater origin hypothesis requires further testing and refinement with additional phylogenetic studies.

*Rhithrogena* Eaton, 1881 (Ephemeroptera, Heptageniidae) is a predominantly Holarctic mayfly genus [42]. Highest *Rhithrogena* species diversity is found in the Palaearctic [43], with a few species in northern Neotropical and Oriental regions [33, 44, 45]. It is the second-most species-rich mayfly genus, with 148 recognized species to date [46], 74 of which have been recorded in Europe [47, 48]. European species are particularly diverse in the Alpine region, with more than 30 described species, half of which are thought to be local endemics. *Rhithrogena* immature stages are often an abundant and diverse component of the benthic invertebrate community, and are routinely used as bio-indicators of water quality [49]. They inhabit most types of running waters, from large floodplain rivers to small and mountainous headwater streams. However, many species are cold-tolerant, with larvae living in fast-flowing, well-oxygenated streams and rivers [43], suggesting that adaptation to such environmental conditions may have played an important role in their diversification in the Alps.

The identification of many European *Rhithrogena* species remains challenging because of a lack of reliable morphological characters in both flying adults and aquatic immature stages (e.g. [43, 50]). A recent study of European species membership using one mitochondrial (mt hereafter) and one nuclear (nuc hereafter) gene fragment highlighted the occurrence of several cryptic species within the genus [51]. Phenotypic plasticity, convergence, or local adaptation could explain such ambiguities (e.g. [52–55]), as could a recent origin of species and incomplete morphological differentiation. *Rhithrogena* species are arranged into several “species groups” [56–58] that are themselves easily identifiable. Although Vuataz et al. [51] recovered five of six studied species groups to be monophyletic, relationships within and among species groups remained unclear.

Here we used two mitochondrial and three nuclear gene regions to reconstruct a species-level phylogeny of European Alpine *Rhithrogena*. Our first aim was to resolve the relationships among *Rhithrogena* species and species groups, where a previous phylogeny showed insufficient resolution. We then determined whether their origin, diversification and changes in speciation rates could be linked to paleoclimatic events, and whether a phylogeny of *Rhithrogena* provides support for a headwater origin hypothesis in the European Alps.

## Methods

### Sampling and sequencing

**Fig. 1:**
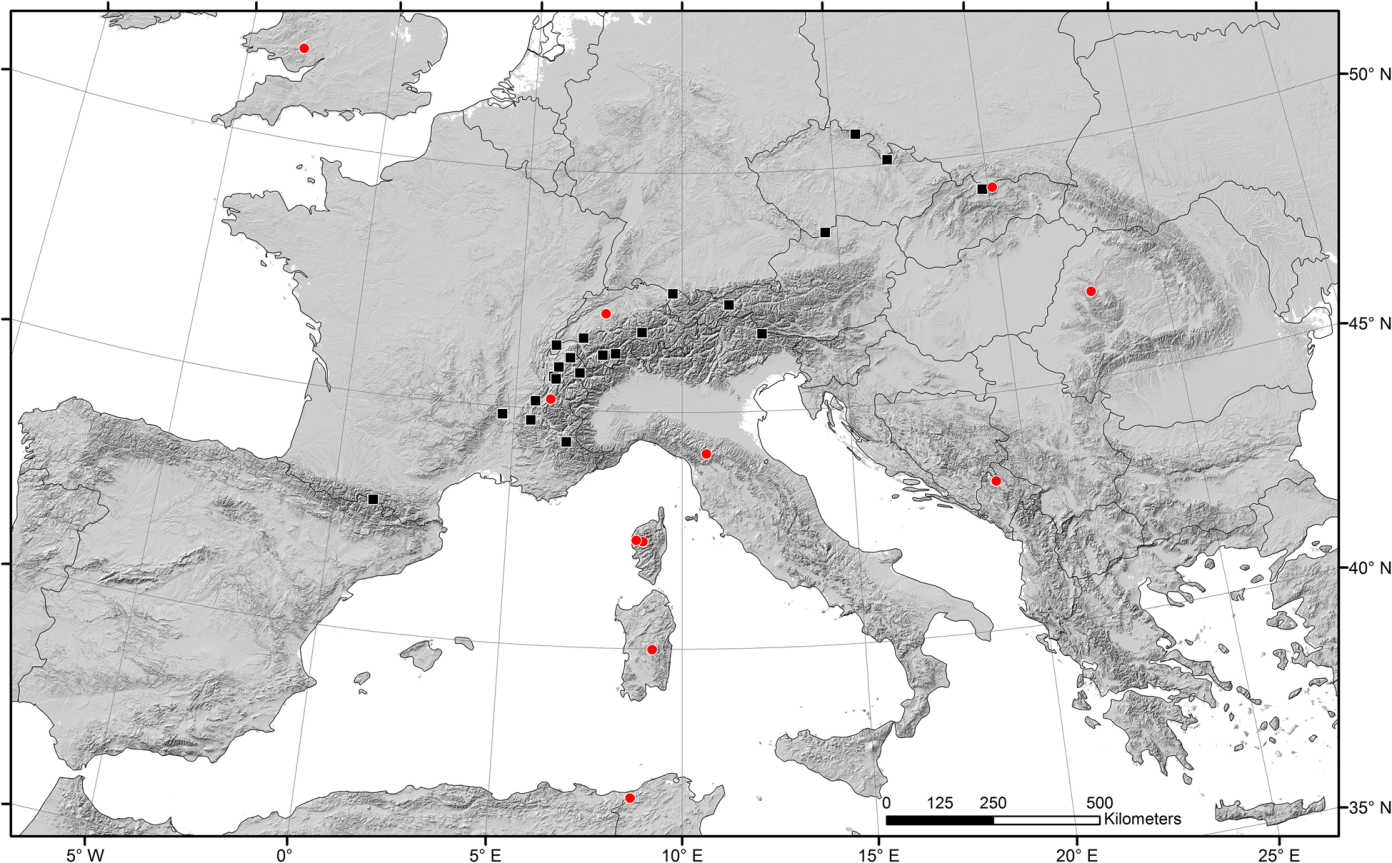
Map of sampling localities from which *Rhithrogena* individuals were collected. *Red circles* indicate newly sampled localities; *black squares* indicate data from Vuataz et al. [51]

Larval *Rhithrogena* were collected from streams between March 2006 and May 2010 at 35 localities in the European Alps (France, Switzerland, Austria, Italy) and from additional localities in the Pyrenees and Corsica (France), the Jura Mountains (Switzerland), the Apennine Mountains and Sardinia (Italy), the Bohemian Forest and the Sudetes Mountains (Czech Republic), the Apuseni Mountains (Romania), the Occidental Carpathians (Poland), the Dinarides (Montenegro), the Cambrian (Wales), and the Kroumir Mountains (Tunisia; Fig. 1). Individuals were preserved in pure ethanol in the field, and stored in the laboratory at **-**20 **°**C in fresh pure ethanol. Ventral-, dorsal- and lateral-view photographs of each individual were taken prior to DNA extraction as described in Vuataz et al. [51]. For phylogenetic analysis, we first chose one representative from each of the 31 GMYC species reported in Vuataz et al. [51]. Of these, 27 were vouchers from Vuataz et al. [51], and four were newly sequenced for this study. Seven additional specimens were then included, based on morphological and mt evidence (data not shown) that they were distinct species not included in Vuataz et al. [51]. In each case, we selected individuals 1) with the most available sequence data in order to obtain a data matrix that was as complete as possible, and 2) sampled from type localities (topotypes) when available (see Additional file 1: Table S1, for a more detailed description of the sampling).

For all individuals, five gene fragments were sequenced for phylogenetic analyses: mt cytochrome c oxidase subunit I (*cox1*) and the large ribosomal subunit 16S rRNA; nuc phosphoenolpyruvate carboxykinase (PEPCK), elongation factor 1-alpha (EF-1α) and wingless (wg). *Cox1* and PEPCK were amplified and sequenced as described in Vuataz et al. [51]. The other genes were amplified and sequenced as described in Vuataz et al. [41].

### Sequence alignment and models of sequence evolution

Forward and reverse sequencing reads were assembled and edited using CodonCode Aligner v. 3.7.1.1 (CodonCode Corporation, Dedham, MA). Heterozygous sites (in nuc genes) were identified as double peaks within the chromatograms and were scored according to the IUPAC code. Sequence alignment was performed using MAFFT [59] as implemented in Jalview v. 2.6.1 [60]. Sequence alignment was straightforward for *cox1*, 16S, EF-1α, wg and the coding regions of PEPCK because there were no insertions or deletions other than a 1-bp indel in the 16S sequences. A length-variable non-coding (intron) PEPCK section was detected using amino acid translation, with boundaries identified following the GT-AG rule (see [51]). A total of 201 bp within the intron could be unambiguously aligned (123 bp at the 5’ end, 14 bp internal and 64 bp at the 3’ end) and were retained for analyses. The remaining intron region was removed from the PEPCK alignment.

Identical haplotypes (*cox1*, 16S) and genotypes (PEPCK, EF-1α, wg) were removed from each alignment using Collapse v. 1.2 [61], and the number of polymorphic and parsimony-informative sites were calculated in MEGA v. 5.03 [62]. The best evolutionary model was selected for each gene fragment (Table 1) following the second-order Akaike information criterion (AICc) implemented in MrAIC v. 1.4.4 [63] under the option using the models implemented in MrBayes. In order to accommodate different substitution rates among codon positions, we used partitioned models of evolution for *cox1* and PEPCK (e.g. [64, 65]). Following the partition scheme from Vuataz et al. [51], we examined *cox1* in two partitions: one with first and second codon positions and one with third positions (1 + 2, 3); for PEPCK, we used one partition with first and second codon positions and a second with third positions and the intron (1 + 2, 3 + intron).

**Table 1:** Sequence variation for each gene region and for the three concatenated matrices (mitochondrial: *cox1* + 16S; nuclear: PEPCK + EF-1α + wg; combined: all five gene fragments combined)

Data set (*n* = 38)	bp	*K*	*S*	*S*_i_	%*S*_i_	Model
*cox1*	658	38	237	217	33	GTR + Γ + I
16S	511	36	67	45	9	GTR + Γ + I
PEPCK	557	37	154	94	17	HKY + Γ
EF-1α	139	24	27	12	9	K2P + Γ
wg	463	27	31	11	2	GTR + I
mitochondrial	1169	38	304	262	22	
nuclear	1147	37	212	120	10	
combined	2316	38	516	383	17	

*n* = number of sequences, bp = size of aligned matrices, *K* = number of haplotypes (*cox1*, 16S) or genotypes (PEPCK, EF-1α, wg) or a combination of both (combined), *S* = number of polymorphic sites, *S*_i_ = number of parsimony-informative sites, Model = best evolutionary model selected following the second-order Akaike information criterion (AICc). Concatenated matrices were examined with partitioned analysis using the best model for each gene fragment (see Methods)

### Tree reconstruction

Our alignments were examined under both coalescent-based species tree and supermatrix (concatenated) approaches. Species tree reconstructions were carried out under a multispecies coalescent framework [66, 67] as implemented in *BEAST v. 2.3.2 [68]. As outgroup for all reconstructions, we used *Cinygmula* McDunnough, 1933, which belongs to the same subfamily as *Rhithrogena* (Rhithrogeninae; see [44]). The species tree reconstructions were performed using the haplotype alignments. For this, the genotypes of the nuclear gene alignments (PEPCK, EF-1α, wg) were phased using the probabilistic Bayesian algorithm implemented in PHASE v. 2.1.1 [69, 70] with a cutoff value of 0.6 [71, 72]. One site originally coded as ‘D’ was implemented as ‘N’. Input and output files were formatted using the scripts from SeqPHASE [73]. Heterozygous sites that could not be resolved were coded as ambiguity codes and remained in the data set for subsequent sequence analyses. All matrices were re-aligned after phasing with MAFFT. The individuals were *a priori* assigned to GMYC species (see Additional file 1: Table S1). As models of sequence evolution, we initially implemented the model combination selected by AICc (see Table 1). Since this specification resulted in poor sampling of several parameters (ESS < 80), even when running up to two billion generations, we used for each partition the model HKY + Γ (sensu [65]). The maximum clade credibility trees produced under both model combinations resulted in very similar topologies, which only silghtly differed in poorly supported terminal nodes (posterior probabilities < 0.4) within the semicolorata group (data not shown). We used a relaxed uncorrelated lognormal clock for gene tree estimation at each locus and a Yule speciation-process prior with two independent runs of 900 million generations with sampling for each every 90,000 generations. The two runs were combined in LogCombiner v. 2.3.2 [68] with a burnin of 10 %, whereby all parameters reached ESS > 200. The maximum clade credibility trees were obtained using TreeAnnotator v. 2.4.1 [68] applying a burnin of 40 %.

For the supermatrix-based approach, Bayesian inference (BI) and maximum likelihood (ML) tree reconstructions were conducted using MrBayes v. 3.1.2 [74] and Garli v. 2.01 [75, 76], respectively. We first performed separate tree searches on mtDNA (*cox1*, 16S) and nDNA (PEPCK, EF-1α, wg). After detecting no major incongruence (see Additional file 2: Figure S1 and Additional file 3: Figure S2), we ran a concatenated analysis using all five gene fragments. In all analyses, each gene fragment was used as a partition in addition to the *cox1* and PEPCK partition scheme defined in the previous section. For each BI analysis, two independent analyses of four MCMC chains were run for 20 million generations sampling trees every 1000 generations. The stationary nucleotide frequencies, alpha shape parameter of the gamma distribution, relative rate of substitution and proportion of invariant sites were unlinked across partitions, and the ratepr command was set to variable (see [77]). Two million generations were discarded as a burnin after visually verifying that likelihood curves had flattened-out and that the independent runs converged using Tracer v. 1.6 [78]. Maximum likelihood phylogenetic inference was performed with 1000 bootstrap replicates, which were summarized in SumTrees v. 4.0.0 [79]. Heuristic searches were used to find the topology with the best likelihood score. Thereby, the searches were conducted using automatic termination, after a maximum of five million generations, or, alternatively, after 10,000 generations without significant (*p* < 0.01) improvement of scoring topology.

### Molecular dating and diversification

**Fig. 2:**
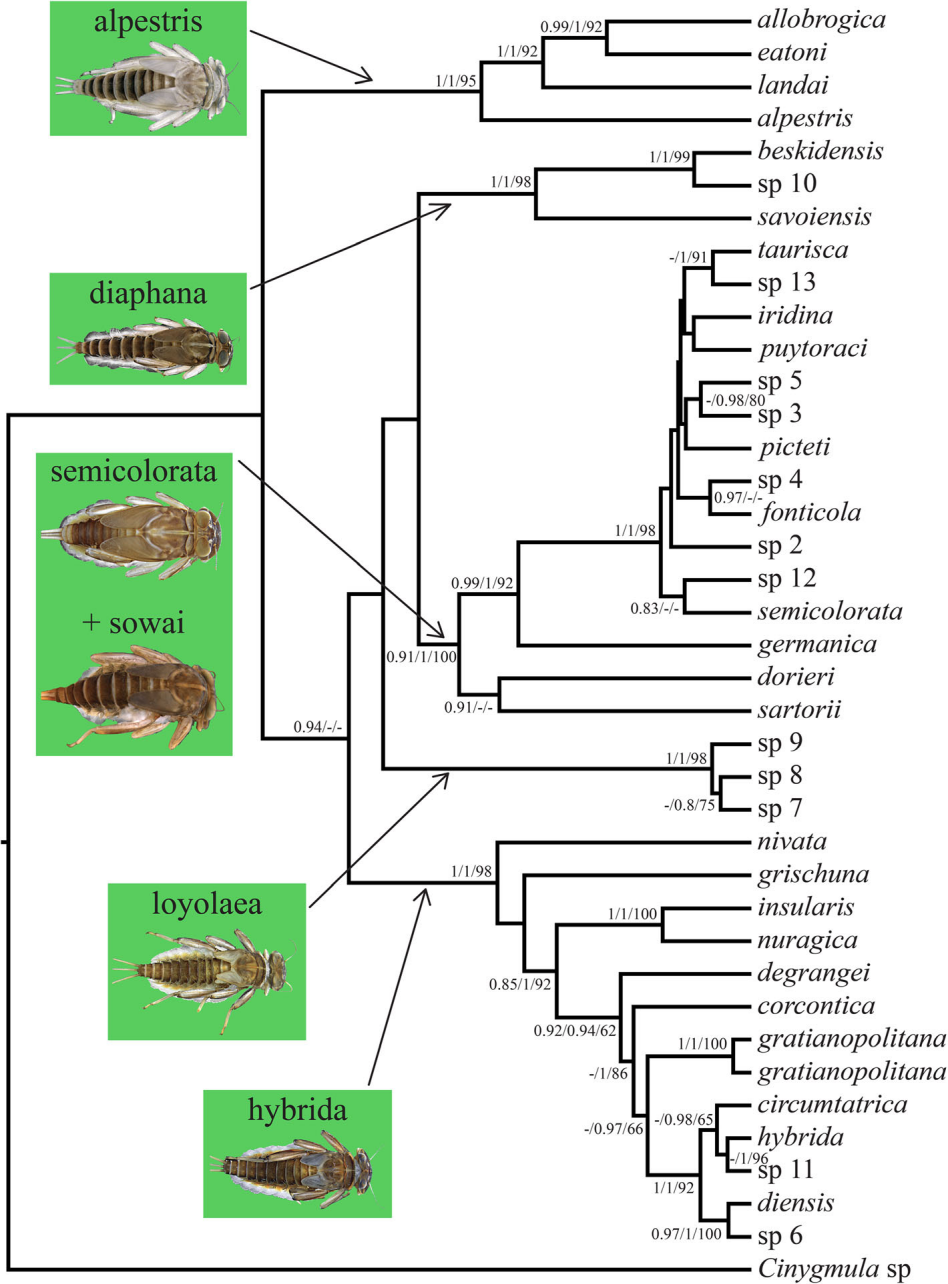
Coalescent-based chronogram (*BEAST maximum clade credibility tree) reconstructed from the five-gene data set. Species groups are indicated with green shading, including a photograph of a species group member. For each node, *BEAST Bayesian posterior probability (*PP), MrBayes Bayesian posterior probability (PP), and maximum likelihood bootstrap support (BS) values are given (*PP/PP/BS) only if posterior probabilities > 0.8 and bootstrap support > 60

**Fig. 3:**
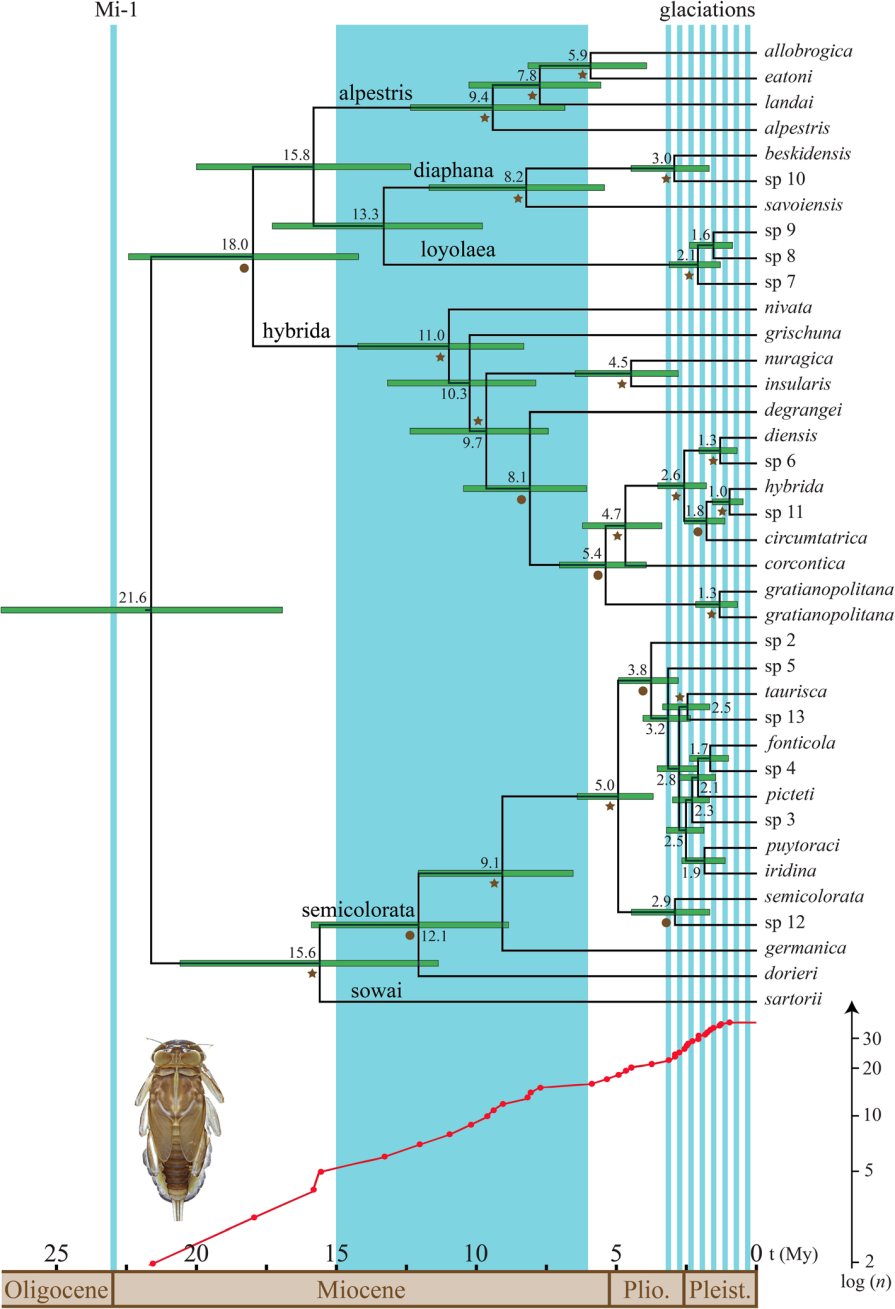
Ultrametric mitochondrial concatenated (*cox1* + 16S) phylogeny (*top*) obtained using a relaxed lognormal molecular clock and a standard insect mutation rate of 0.0115 substitution/site/My in BEAST, and the corresponding LTT plot of the empirical data (*bottom*), with vertical axis (number of GMYC species) in logarithmic scale. For each node, estimated ages (horizontal axis, time before present in My) are given. *Horizontal green bars* indicate the 95 % highest posterior density interval. *Stars* indicate lineages that were constrained to be monophyletic according to the mt + nuc concatenated phylogeny (i.e. PP > 0.95 and BS > 80; see Fig. 2 and Additional file 4: Figure S3); *circles* indicate PP > 0.9. The *blue zone* represents a period of global climatic cooling trend, *empty zones* warming trends, following Zachos et al. [109]. *Blue vertical bars* symbolize the Oligocene/Miocene boundary glacial maximum (Mi-1; 23 Mya) and the Quaternary glacial cycles (glaciations; 3.2 Mya to present). Species groups are specified above corresponding branches

We chose a commonly used mt mutation rate of 0.0115 substitutions per site per million years (My hereafter), equivalent to 2.3 % divergence per My [80], to date diversification events. This is because no fossils, geological/paleoclimatic events or independent studies could be used to calibrate tree nodes for *Rhithrogena*. This widely used rate (see [81]) was calculated from studies of arthropods and is based on both protein-coding and ribosomal mt genes. Our concatenated mt data set (*cox1* + 16S) was used for branch-length estimation using a Bayesian relaxed (uncorrelated lognormal) molecular clock approach implemented in BEAST v. 1.8.3 [82]. We initially used a GTR + Γ + I model of evolution for each partition, as selected by AICc (see Table 1). Since this specification resulted in poor sampling of several parameters (ESS < 50), we used a HKY + Γ + I model for each partition, which was the second-best model according to AICc (data not shown). A Yule process was preferred because it provides useful tree priors for species-level studies [83]. The clock models and the trees were linked for the concatenated mt data set. The substitution models of evolution were implemented separately (i.e. unlinked) for each of the three partitions (*cox1* codon position 1 + 2; *cox1* codon position 3; 16S), and substitution rate parameters and base frequencies were unlinked across *cox1* partitions. All base frequencies were estimated from the data, the number of gamma categories was set to six, and a random starting tree was used. Well-supported nodes (BI posterior probabilitiy (PP) > 0.95 and ML bootstrap support (BS) > 80) of the concatenated mt phylogeny were constrained as monophyletic (18 constrained nodes; see Fig. 3 and Additional file 4: Figure S3). For node constraints, we favored the concatenated over the coalescent-base phylogeny, because it was the best supported phylogeny overall (see Results and Fig. 2). All other options and priors were set to default. Two independent MCMC chains were run for 100 million generations and sampled every 1000 generations, resulting in 100,000 trees for each run. Run convergence was verified using Tracer (as above) and the first 10,000 trees were then discarded from each run as a burnin, whereby all parameters reached ESS > 2000. Log and tree files were combined using LogCombiner v. 1.8.3 [82], resulting in 180,000 trees in the combined analyses. The maximum clade credibility tree was built with TreeAnnotator v. 1.8.3 [82] with all options set to default.

The diversification rate of *Rhithrogena* lineages was examined using γ-statistics [84] and a birth-death likelihood (BDL) analysis [85]. An important problem with empirical diversification analyses is incomplete taxon sampling, which could lead to a variety of misinterpretations of phylogenies (e.g. [86–88]). One recent advance for handling missing species is the CorSiM approach [89, 90], which is based on the simulation of missing splits under speciation/extinction models. To correct for incomplete sampling in our empirical *Rhithrogena* phylogeny, we used the CorSiM approach to simulate 1000 trees with 36 additional (i.e., missing) species. This brought the total to 74 species, which corresponds to the currently accepted level of species diversity of the genus in Europe [47, 48]. We assumed a random sampling (sensu [90]) because we focused on Alpine species rather than selected species from each species group. The γ-statistic was calculated using the function gamStat in the laser v. 2.4-0 [85] package for R v. 3 [91]. Values of γ > 0 indicate an increasing number of nodes toward the tips of the phylogenetic tree. The BDL analysis with two constant-rate models (Yule, birth-death) and three variable-rate models (logistic density-dependence, exponential density-dependence, two-rate variant of the Yule model) was performed using the function fitdAICrc in laser.

### Ancestral state reconstruction

To test whether European *Rhithrogena* fit the headwater origin hypothesis with subsequent downstream diversification, we reconstructed the ancestral altitude range of the two species groups hybrida and alpestris using the current species distributions as raw data. The other species groups were not included in the ancestral state analyses for specific reasons: the semicolorata group phylogeny was poorly resolved (see Results), the diaphana group is mainly Mediterranean and thus most species were not included in our Alpine-oriented sampling, and all members of the loyolaea group live exclusively at high elevation. In the absence of published altitude ranges of *Rhithrogena* species at the European scale, we used our own extensive sampling (including unpublished data) to assign elevational distributions. For each species, we considered our minimum and maximum altitude records as a proxy for actual altitude ranges (Additional file 1: Table S3). Subtrees of the hybrida and alpestris species groups were extracted from both our species tree chronogram (coalescent-based approach), and concatenated ML phylogram (supermatrix approach). We then performed residual maximum likelihood (REML) ancestral state reconstructions [92] of elevation on both topologies using the ace function of the ape [93] package for R, which assumes a Brownian motion model of character evolution implemented for continuous variables.

## Results

### Data set characteristics

The concatenated data matrix of five gene fragments was > 99 % complete. There were no missing data in the *cox1*, 16S and wg matrices. Missing PEPCK data resulted from seven sequences that lacked 3’ and/or 5’ ends or a small intron section (261 missing characters in the matrix). There was a single EF-1α sequence for which a small internal section of 11 bp was ambiguous. A total of 103 nucleotides in the nuc data set were heterozygous. The concatenated mt data set contained 59 % of the total variation, primarily found in the third codon positions of the *cox1* fragment (68 % of the mt variation). Most of the nuc variation (68 %) was found in the third codon positions and in the intron section of PEPCK. The percentage of parsimony-informative sites within each gene fragment ranged from 33 % (*cox1*) to 2 % (wg; Table 1). Two individuals had identical nuc genotypes (voucher 110FRSAGT and 111FRSAGT; Additional file 1: Table S1), whereas all individuals had distinct mt haplotypes.

### Phylogenetic reconstruction

All four reconstructions, namely the mt + nuc coalescent-based (Fig. 2), the mt concatenated (Additional file 2: Figure S1), the nuc concatenated (Additional file 3: Figure S2), and the mt + nuc concatenated (Additional file 4: Figure S3), produced five well supported monophyletic species groups: (1) alpestris, (2) diaphana, (3) semicolorata + *Rh. germanica* + *Rh. sartorii*, (4) loyolaea, and (5) hybrida + hercynia + *Rh. insularis* + *Rh. nuragica*. The only variation occurred in the placement of *Rh. sartorii* from Tunisia, the sole representative of the sowai species group included here (see Discussion). In the coalescent-based tree, *Rh. sartorii* + *Rh. dorieri* formed a sister clade to the semicolorata species group. In the mt concatenated tree, *Rh. sartorii* was sister to the semicolorata species group. In the other reconstructions, its placement was equivocal. The alpestris species group was sister clade to the others in the mt + nuc coalescent-based tree, but all other reconstructions did not resolve relationships among the species groups.

Species-level relationships within the alpestris, diaphana and loyolaea species groups were fully congruent in all reconstructions, with increased node support in the mt + nuc coalescent-based and mt + nuc concatenated reconstructions compared to the mt and nuc concatenated reconstructions. Species-level relationships within the semicolorata species group were generally poorly supported in all reconstructions, but with *Rh. germanica*, *Rh. dorieri* and *Rh. sartorii* well supported as sister taxa to the other species group members in all reconstructions. Species-level relationships within the hybrida species group were weak overall in the mt + nuc coalescent-based, in the mt concatenated and in the nuc concatenated reconstructions, but were fully resolved and well supported in the mt + nuc concatenated reconstruction; the only exception was the equivocal relationships between *Rh. nivata* and *Rh. dorieri*, well supported as sister taxa to the other species group members.

### Dating and diversification

The root of our phylogenetic tree was dated at 21.6 million years ago (Mya hereafter), with a 95 % highest posterior density interval (95 % HPD hereafter) of 27.0 – 16.9 Mya. This node age roughly corresponds to the Oligocene-Miocene boundary (Fig. 3). Most species group diversifications occurred in the middle and late Miocene: semicolorata (12.1 Mya; 95 % HPD 15.9 – 8.9 Mya), hybrida (11.0 Mya; 95 % HPD 14.3 – 8.3 Mya), alpestris (9.4 Mya; 95 % HPD 12.4 – 6.9 Mya), and diaphana (8.2 Mya; 95 % HPD 11.7 – 5.4 Mya). The loyolaea species group diversification appeared notably younger, dated at 2.1 Mya (95 % HPD 3.1 – 1.3 Mya), in the Pleistocene. Examined globally, 17 of 37 (46 %) speciation events (i.e., nodes) occurred between 3.1 Mya (late Pliocene) and 1.0 Mya (early Pleistocene), broadly overlapping the Quaternary glaciations (Fig. 3). Terminal nodes from this recent diversification include *Rh. diensis*, *Rh. hybrida*, *Rh. circumtatrica*, *Rh. gratianopolitana*, *Rh. beskidensis*, *Rh. semicolorata*, *Rh. taurisca*, *Rh. puytoraci*, *Rh. iridina*, *Rh. picteti*, and *Rh. fonticola.*

**Fig. 4:**
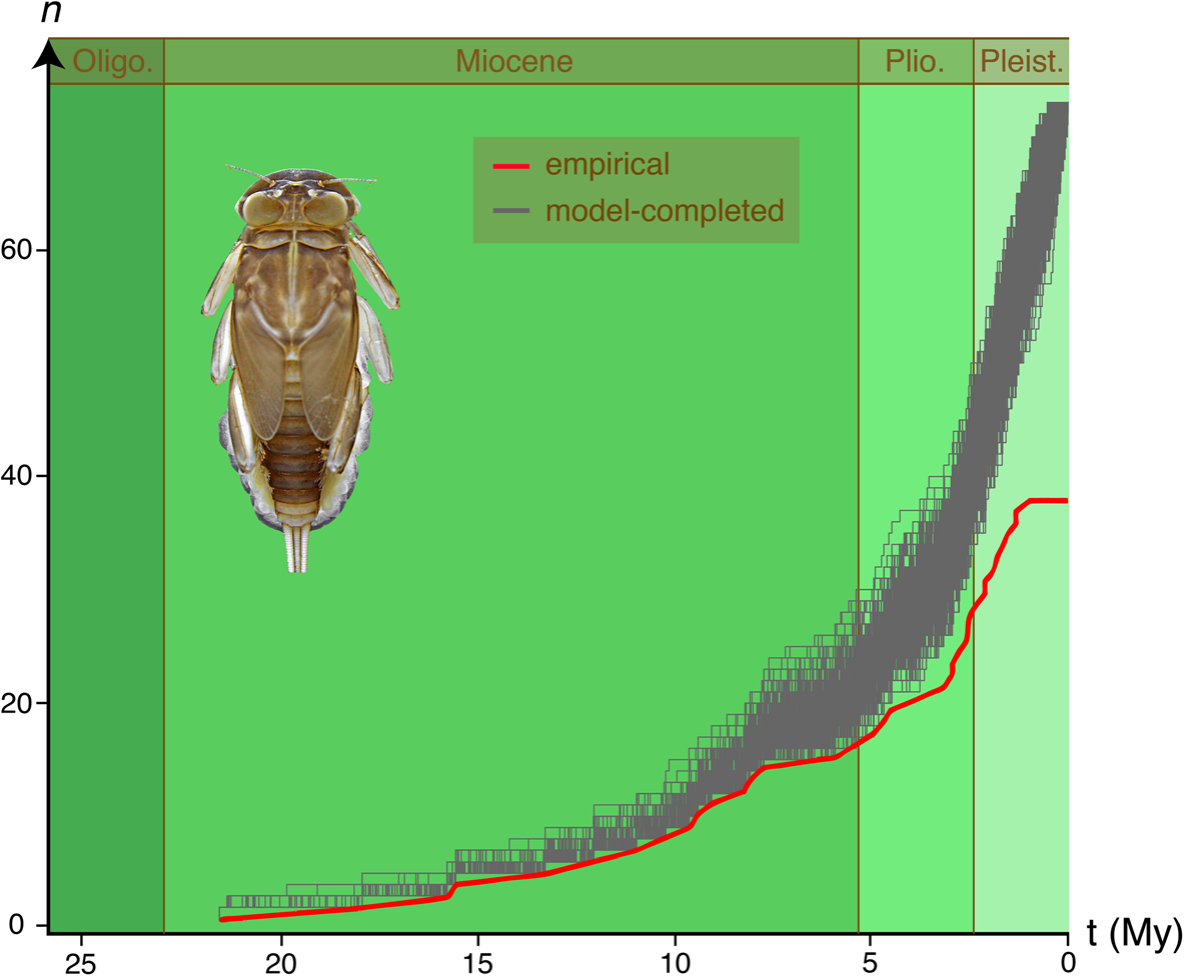
LTT plots of the European *Rhithrogena*, showing the number of GMYC species (*n*) versus time before present (t, in My). *Red line* corresponds to the empirical data (BEAST mitochondrial concatenated (*cox1* + 16S) incomplete phylogeny), *grey lines* to the 1000 simulated complete phylogenies

**Fig. 5:**
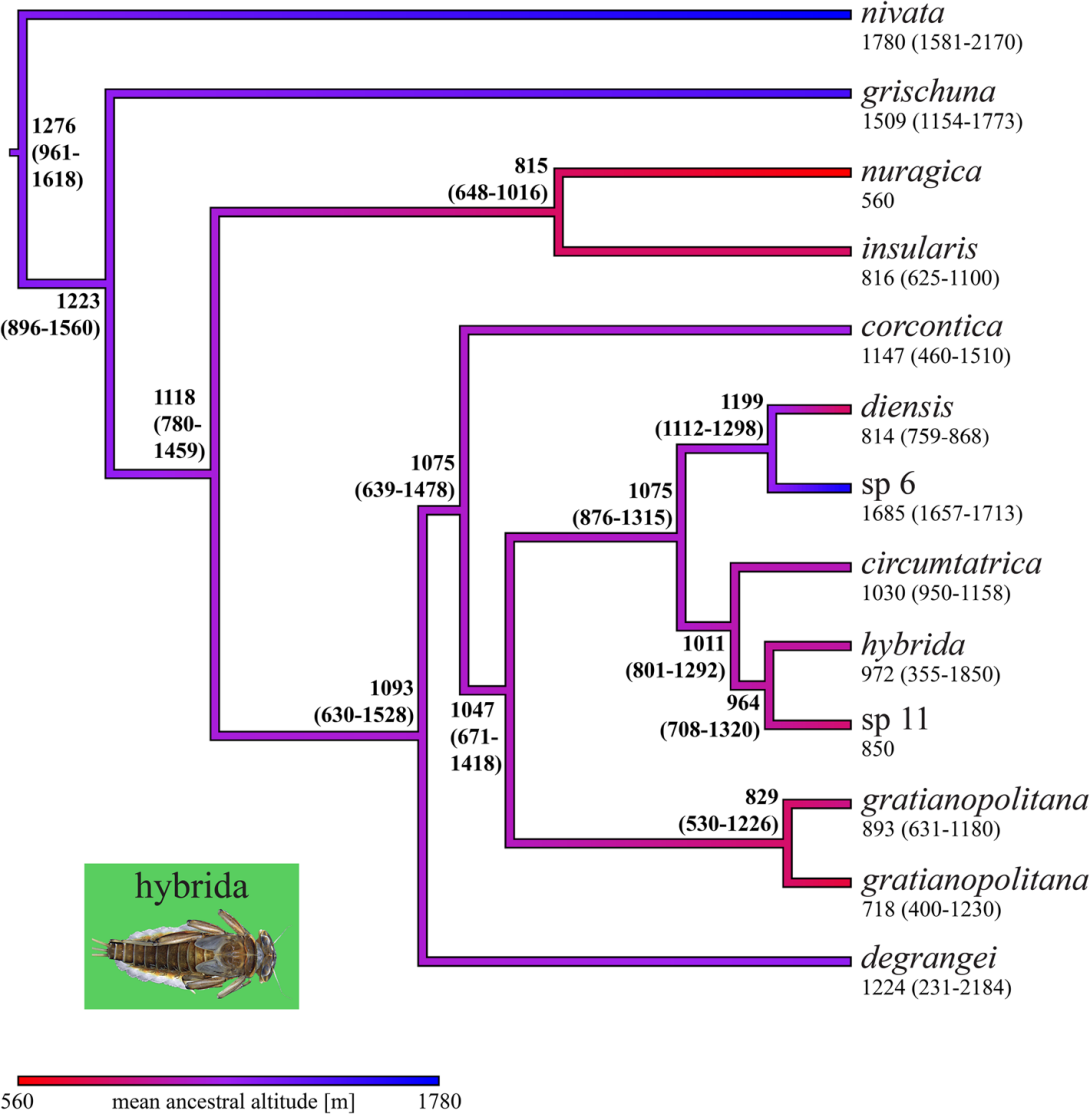
Coalescent-based chronogram (*BEAST maximum clade credibility tree) of the hybrida species group extracted from the global five-gene species tree, with ancestral altitudes reconstructed using the REML method. Mean ancestral altitudes (with minimum and maximum ancestral altitudes between brackets) are indicated for each node. Mean altitudes (with altitude ranges between brackets) extracted from authors’ own records (see Additional file 1: Table S3), and used as raw data for the ancestral altitude reconstruction, are given below each terminal GMYC species. The color gradient symbolizing the mean altitude gradient along the tree branches was obtained using the contMap function of the phytools package [129] for R

Speciation and extinction rates calculated using TreePar (bd.shifts.optim() function) and used for tree simulations with CorSiM were λ = 0.24 and μ = 0.08. The data set completed by simulation (Fig. 4) was characterised by γ = 1.07 (SD 0.44, one-tailed *p* = 0.82), indicating that rate constancy was not rejected. The BDL analyses indicated a preference for a Yule-2-rates model in 62 % of data sets (AIC -113.21; SD 7.92; Additional file 1: Table S4), with a decrease in the diversification rate at 0.22 Mya, corresponding to the most recent branching events in the clock-constrained tree (Fig. 4). A Yule (pure birth) model was preferred in 33 % of data sets (AIC -112.18; SD 6.73) and a birth-death model in 6 % of data sets (AIC -111.84; SD 7.78). The empirical LTT plot of the mt ultrametric tree suggested an increase in speciation rate curve occurring from ca. 3.0 to 1.0 Mya in the late Pliocene and early Pleistocene (Figs. 5, bottom and 6). The pattern departure of simulated (74-taxon) trees from the empirical (38-taxon) tree suggested that deeper nodes were oversampled in our empirical tree (see Fig. 1 in [90]).

**Fig. 6:**
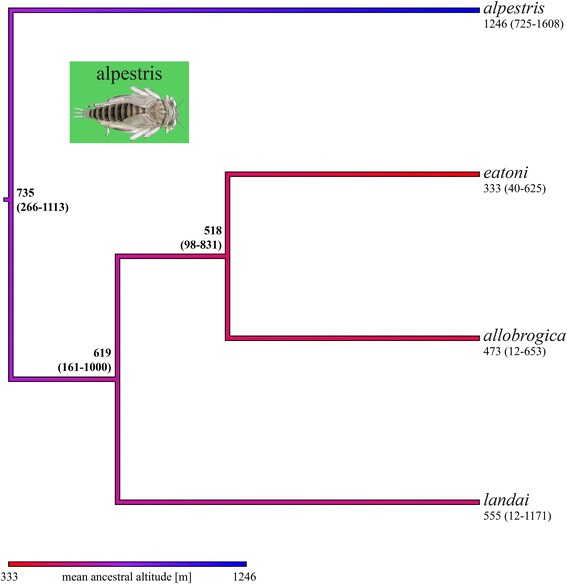
Coalescent-based chronogram (*BEAST maximum clade credibility tree) of the alpestris species group extracted from the global five-gene species tree, with ancestral altitudes reconstructed using the REML method. The altitudes and the color gradient are indicated as in Fig. 5

### Ancestral state reconstruction

The ancestral state reconstructions of elevation indicated a trend of decreasing altitude ranges toward the present for the species tree chronograms and for the concatenated ML phylograms in the hybrida and alpestris species groups. For the ancestral species of the hybrida group, the species tree chronogram (Fig. 5) indicated a mean altitude of 1276 m (range 961–1618 m), and the concatenated ML phylogram (Additional file 5: Figure S4) a mean altitude of 1343 m (range 966–1732 m), suggesting an origin of the group in mid to high-elevation streams. For the ancestral species of the alpestris group (Fig. 6), the species tree chronogram indicated a mean altitude of 735 m (range 266–1113 m), and the concatenated ML phylogram (Additional file 6: Figure S5) a mean altitude of 846 m (range 365–1233 m), suggesting an origin of the group in low to mid-elevation streams.

## Discussion

### Species groups

Several species groups within European *Rhithrogena* have been proposed based on larval and adult morphology (e.g. [56, 57, 94]). The alpestris, diaphana, loyolaea, semicolorata (including germanica) and hybrida (including hercynia) groups were recently recovered as monophyletic entities in independent mt (*cox1*) and nuc (PEPCK) gene tree reconstructions [51]. Based on additional gene fragments, increased taxon sampling (Additional file 1: Table S1), and a coalescent-based approach applied here, these findings were confirmed. We also included *Rh. insularis* from Corsica and *Rh. nuragica* from Sardinia, both members of an additional putative species group, insularis [95], but both were recovered here within the hybrida species group. The only other genetic analysis to date [58] used allozymes and a genetic-distance tree to divide *Rhithrogena* into only two species groups. Our study included most species from the Zurwerra et al. [58] study and while broadly congruent, notably within hybrida and semicolorata species groups, there were important differences. Specifically, Zurwerra et al. [58] grouped members of alpestris and hybrida with the loyolaea species group, as well as semicolorata with diaphana, whereas we found clear evidence for their separation.

*Rhithrogena sartorii*, recently described from Tunisia [96], was thought to share morphological characteristics with the insularis species group [96], although a more recent examination of mounted specimens and different characters, including the shape of the first gill plica and the spines on the upper face of the femora, related this species to members of the sowai species group (M. Sartori, unpublished data). This group is well diversified in the Mediterranean area, particularly the Iberian and Balkan peninsulas [97, 98], indicating *Rh. sartorii* is a marginal representative. Our mt concatenated phylogenies recovered *Rh. sartorii* as sister taxon to the semicolorata species group, whereas the other reconstructions included it in the semicolorata group or were inconclusive. A phylogenetic analysis including more members of the sowai species group would help to determine whether the group is monophyletic, and whether it should be fused with semicolorata.

### Timing of diversification

For molecular dating, we used a previously estimated evolution rate of mtDNA nucleotide substitution [80] because reliable estimates of node ages are difficult to obtain for mayflies. The only known fossils of *Rhithrogena* were described by Demoulin [99] from Baltic Amber (40 – 50 Mya; e.g. [100]); however, their attribution to *Rhithrogena* was contested by Kluge [101], who noted that Heptageniidae genera are based on larval characteristics rather than the adult features used by Demoulin [99]. Geological events, such as island formation, are also problematic. Our analysis revealed sister taxa on neighboring islands (*Rh. insularis* on Corsica and *Rh. nuragica* on Sardinia), but using the separation of the Corso-Sardinian microplate from the continent or the separation of Sardinia from Corsica as calibration points is potentially problematic. There is strong evidence for overseas dispersal in the two mayfly families examined to date: Heptageniidae [41] and Baetidae [102, 103]. Although there is evidence for diverging rates between lineages (e.g. [104–106]), the commonly used mtDNA rate we selected was broadly supported by Papadopoulou et al. [81] in a study of tenebrionid beetles using a number of geological calibration points from the mid-Aegean trench.

Our empirical tree indicated an increase in *Rhithrogena* speciation rate from ca. 3.0 to 1.0 Mya, corresponding to the late Pliocene and early Pleistocene. This period of time largely overlaps the Quaternary glacial cycles, which are well known for having promoted lineage divergence and speciation in a variety of organisms (see [6] for a review). In contrast, our analysis using simulated trees to account for incomplete sampling of *Rhithrogena* species detected a significant reduction in diversification rate at 0.22 Mya (Additional file 1: Table S4, and Fig. 4). This shift potentially results from the absence of intraspecific variation in our sampling (e.g. [86, 107]), which included a single individual per GMYC species. Alternatively, this shift could be due to a lack of recent species in our sampling, either because they occur in regions poorly coverred by our sampling like the Iberian and Italian peninsulas, or because the *cox1* failed to detect the most recent speciation events. Rather than any increase in diversification, the simulation approach indicated a relatively steady increase in the number of lineages over time, suggesting that speciation is relatively constant and ongoing, and that glacial cycles have not been disproportionately important. Nonetheless, more than 50 % of the sampled species arose during the 3.0 – 1.5 Mya period in the empirical tree, regardless of whether speciation rate varied significantly. Species that recently arose include all members of the loyolaea species group, most of the semicolorata species group, and some members of the hybrida and diaphana species groups. A recent divergence between these species may partly explain the difficulty of finding reliable morphological characters for *Rhithrogena* species identification [43, 50].

Interestingly, the origin of European *Rhithrogena* roughly corresponded to the Oligocene/Miocene boundary (ca. 23 Mya), which coincided with one of the three major climatic aberrations of the Cenozoic: the Oligocene/Miocene glacial maximum [108, 109]. This deep glaciation spanning 0.2 My, followed by a series of smaller glaciations, which happened in a global context of climate warming, promoted rates of turnover and speciation in many groups, including mammals, birds, ostracods, and planktonic organisms [110–112]. Similar turnovers in a number of groups (e.g. [113–116]) were also linked to another major climatic aberration, the older Eocene/Oligocene glacial period [108, 109].

The Messinian Salinity Crisis (MSC, 5.96 – 5.33 Mya), a geological event that had considerable impact on the Mediterranean flora and fauna (e.g. [117, 118]), potentially promoted freshwater invertebrate dispersal between Mediterranean islands, with subsequent population isolation after the rapid refilling of the Mediterranean Sea [119]. A recent *cox1*-based study [120] found that genetic differentiation of several continental and insular mayfly populations coincided with the MSC. The origin of the three insular endemic species included in our sampling also largely overlapped the MSC period: *Rh. eatoni* from Corsica (5.9 Mya; 95 % HPD 8.2 – 3.9), *Rh. insularis* from Corsica and *Rh. nuragica* from Sardinia (4.5 Mya; 95 % HPD 6.5 – 2.8), thus supporting the important role of this geological event in the speciation of Corso-Sardinian mayflies.

Using the currently recognized taxonomy would have resulted in species over-splitting because single GMYC species have up to four names (see [51]). Our use of GMYC species (see Material and Methods) was intended to minimize this problem. However, Vuataz et al. [51] defined four cases of potential over-splitting in the delimited GMYC species (involving *Rh. gratianopolitana*, sp 4, sp 6 and sp 10; Additional file 1: Table S1), which if correct would still leave ca. 50 % of species having diversified 3.0 – 1.0 Mya. Although several low elevation species from the Iberian and Italian peninsulas were not sampled here, most central European species are represented. Our Alpine sampling was extensive and included all described species, most of them from their type localities. We can thus be confident that our sampling provides a very good representation of the Alpine diversity.

Additional evidence for the accuracy of our GMYC species comes from the present-day distribution of sampled species. Based on our sampling in the Alps and other mountain chains in Europe (data not shown), several closely related *Rhithrogena *species have distinct or adjacent (i.e., allopatric or parapatric) distribution ranges. Based on our dating analysis, these species diverged from one another during the Pleistocene. Within the hybrida species group, the individuals from one well-supported clade (*Rh. circumtatrica* + *Rh. hybrida* + sp 11 + *Rh. diensis* + sp 6; Fig. 2) are distributed in the Apennines (sp 11), in a small area in the western Alps (*Rh. diensis*, sp 6), in the Carpathians (*Rh. circumtatrica*) and widely in the Alps (*Rh. hybrida*). Within the well-supported loyolaea species group (Fig. 2), sp 7 occured in the Carpathians and the eastern Alps, sp 8 in the central Alps, and sp 9 in the western Alps and the Pyrenees. Such present-day allopatric or parapatric distribution ranges are typically observed in closely related species or populations that diverged in separate refugia during the Quaternary glacial periods (e.g. [7, 121]).

### Ancestral habitat

The headwater origin hypothesis proposes an ancestral position of cold-adapted aquatic insect lineages in headwaters and subsequent diversification into warmer, larger rivers downstream [35–37]. Recently, Statzner et al. [38] and Statzner & Doledec [39] found evidence to support this hypothesis using an analysis of mtDNA and morphological characters from nine *Hydropsyche* (Trichoptera) species along the Loire River (ca. 0 – 1300 m), although node support was partly inconclusive. In contrast, Vuataz et al. [41] found evidence that lowland mayflies (Heptageniidae) diversified to more upstream (colder) areas in Madagascar, while Toussaint et al. [40] reported a complex combination of upstream and downstream diversification events in *Exocelina* diving beetles (Coleoptera, Dytiscidae) in Papua New Guinea.

Our ancestral state reconstructions of elevation constitute the first attempt for mayflies of which we are aware, and provide additional corroboration for the original hypothesis of headwater origins. However, our findings constitute only a preliminary estimate of ancestral aquatic habitats, being derived from a phylogeny of ca. 50 % of the European diversity, and because our own sampling sites served as a proxy of current altitude ranges of *Rhithrogena* species. Two future improvements would verify our findings. First, including more species, particularly low-elevation species from the Iberian and Italian peninsulas that are lacking in our Alpine-oriented sampling, would produce more complete phylogenies. Second, collecting more altitude data at a broader scale, particularly for rare species that we could sample from only one or a few localities, would improve the accuracy of the altitude ranges.

Interestingly, *Rh. nivata* and *Rh. grischuna* were the sister species to all other hybrida group members (Fig. 2), and live at higher elevations than all of them. The distribution of *Rh. nivata* ranges from 1100 to 2400 m in Switzerland [122] and 1160 to 1650 m in France [123]. *Rh. grischuna*, which is thought to inhabit small to medium rivers, was only found above 1150 m in our extensive sampling. In contrast, most other hybrida species group members exploit a wide altitudinal range (*Rh. degrangei*: 200 – 2200 m; *Rh. gratianopolitana*: 400 – 1200 m; *Rh. hybrida*: 400 – 1900 m; *Rh. corcontica*: 500 – 1500 m), or were sampled from lower elevations than *Rh. grischuna*. Similarly, the sister species to all other alpestris group members, *Rh. alpestris*, occurs at a higher elevation than any other member of the species group, and immature stages inhabit headwater streams and small to medium rivers between 500 and 2300 m (greatest occurrence between 1000 and 1800 m) in Switzerland [122] and from 1715 to 2020 m in France [123]. In contrast, *Rh. landai* (including *Rh. vaillanti*; see [51]) larvae inhabit only medium to large rivers between 400 and 700 m in Switzerland [122], although our sampling included three occurences between 1000 and 1200 m; *Rh. allobrogica* lives between 210 and 715 m [124] and *Rh. eatoni* lives exclusively in Corsica from 18 to 1100 m [123]. The loyolaea species group is restricted to cold-adapted species living in high elevation streams (mostly between 1700 and 2200 m; [122]), thus their ancestor was also probably cold adapted. It appears that the diversification of this species group is more recent (Fig. 3) and that there has been no diversification into lower elevations. The semicolorata species group members were sampled at wide altitudinal ranges, with the notable exception of *Rh. germanica*, an inhabitant of large floodplain rivers (low elevations) that has become very rare [125]. The diaphana species group is mainly composed of Mediterranean species [94, 126, 127], the Alpine ones included in our study being marginal representatives.

## Conclusions

Our analysis of two mitochondrial and three nuclear gene regions, combined with an increased taxon sampling compared to previous studies, provided greater resolution and clearer definitions of species groups within European *Rhithrogena*. While our coalescent-based and concatenated phylogenies produced very similar topologies, node supports from the concatenated approach were repeatedly higher, particularly within the hybrida species group. The simulation approach of estimating diversification rates indicated that European *Rhithrogena* speciation is constant and ongoing, with the more recent half of the speciation events overlapping the Quaternary glaciations (<3 Mya). The recent origin and ongoing diversification potentially explain the morphologically cryptic characteristics of many European *Rhithrogena* species. The previously proposed headwater origin hypothesis with subsequent downstream diversification was supported by ancestral state reconstructions in the hybrida and alpestris species groups, indicating speciation has proceeded in part via diversification into warmer and larger rivers downstream. Testing whether this is a consistent pattern in Alpine aquatic insects requires additional analyses of a broader range of lineages.

## Additional files

Additional file 1: Tables S1-S4. (PDF 133 kb) Additional file 2: Figure S1. Bayesian majority-rule consensus tree reconstructed from the concatenated mitochondrial (*cox1* + 16S; 1169 bp) data set. Species groups are indicated with green shading, including a photograph of a species group member. For each node, Bayesian posterior probability (PP) and maximum likelihood bootstrap support (BS) values are given (PP/BS) if PP > 0.8 and BS > 60. (TIF 7398 kb) Additional file 3: Figure S2. Bayesian majority-rule consensus tree reconstructed from the concatenated nuclear (PEPCK + EF-1α + wg; 1147 bp) data set. Species groups and node support are indicated as in Additional file 2: Figure S1. (TIF 7255 kb) Additional file 4: Figure S3. Bayesian majority-rule consensus tree of the mitochondrial + nuclear concatenated (2316 bp) data set. Species groups and node support are indicated as in Additional file 2: Figure S1. (TIF 7566 kb) Additional file 5: Figure S4. ML tree of the hybrida species group extracted from the concatenated (five-gene supermatrix) ML phylogram, with ancestral altitudes reconstructed using the REML method. Mean ancestral altitudes (with minimum and maximum ancestral altitudes between brackets) are indicated for each node. Mean altitudes (with altitude ranges between brackets) extracted from authors’ own records (see Additional file 1: Table S3), and used as raw data for the ancestral altitude reconstruction, are given below each terminal GMYC species. The color gradient symbolizing the mean altitude gradient along the tree branches was obtained using the contMap function of the phytools package [129] for R. (TIF 3301 kb) Additional file 6: Figure S5. ML tree of the alpestris species group extracted from the concatenated (five-gene supermatrix) ML phylogram, with ancestral altitudes reconstructed using the REML method. The altitudes and the color gradient are indicated as in Additional file 5: Figure S4. (TIF 2765 kb)

## Abbreviations

AIC: Akaike information criterion
AICc: Second-order Akaike information criterion
BDL: Birth-death likelihood
BI: Bayesian inference
bp: Base pair
BS: Bootstrap support
*cox1*: Cytochrome c oxidase subunit I
EF-1α: Elongation factor 1-alpha
ESS: Effective sample size
GMYC species: Species delimited using the general mixed Yule-coalescent model
HPD: Highest posterior density interval
LTT: Lineage-through-time
Mi-1: Oligocene/Miocene boundary glacial maximum
ML: Maximum likelihood
MSC: Messinian Salinity Crisis
mt: Mitochondrial
My: Million years
Mya: Million years ago
nuc: Nuclear
PEPCK: Phosphoenolpyruvate carboxykinase
PP: Posterior probability
REML: Residual maximum likelihood
rRNA: Ribosomal ribonucleic acid
SD: Standard deviation
wg: Wingless
