# Supplementary material for: Molecular phylogeny and timing of diversification in Alpine Rhithrogena (Ephemeroptera: Heptageniidae)
Source: BMC Evol Biol. 2016 Sep 21;16:194. doi: 10.1186/s12862-016-0758-1 (PMC5031269; doi:10.1186/s12862-016-0758-1)
Supplement: Additional file 1: Tables S1-S4. — (PDF 133 kb) [file 12862_2016_758_MOESM1_ESM.pdf]

Table S1. Detailed sampling. For each *Rhithrogena* individual, the voucher name (voucher), the origin (country and region), the coordinates of the sampling points (latitude and longitude in decimal degrees), the species group as defined in Figs. 2, 3 (sp group), the GMYC species identification as labeled in Figs. 2, 3 (sp) and the GMYC species number (nb) from Vuataz et al. [1] are indicated. Individuals are named only when they originated from type localities (i.e., topotypes) or could be unambiguously identified, and are otherwise referred to as "sp". For the 11 newly collected individuals (bold), the GMYC species numbers are indicated when they obviously clustered with one of the 31 GMYC species from Vuataz et al. [1]. When individuals are from GMYC species linked to more than one name in Vuataz et al. [1], the latest name(s) are mentioned between brackets.

voucher	country	region	lat	long	sp group	sp	nb
033FRDRDI	France	Drôme	44.746	5.503	hybrida	*Rh. diensis* Sowa & Degrange, 1987	20
037AUTYRO	Austria	Tyrol	47.247	11.472	semicolorata	*Rh. taurisca* Bauernfeind, 1992<br>(*Rh. rolandi* Weichselbaumer, 1995)	5
041CHGRGS	Switzerland	Graubünden	46.674	8.761	hybrida	*Rh. grischuna* Sartori & Oswald, 1988	15
042FRHSAB	France	Haute-Savoie	46.114	6.553	alpestris	*Rh. allobrogica* Sowa & Degrange, 1987	26
045PLPPCI	Poland	Lesser Poland	49.266	19.870	hybrida	*Rh. circumtatrica* Sowa & Soldán, 1986	18
047FRISMA	France	Isère	45.154	5.606	hybrida	*Rh. hybrida* Eaton, 1885<br>(*Rh. austriaca* Sowa & Weichselbaumer, 1988)<br>(*Rh. endenensis* Metzler, Tomka & Zurwerra, 1985)<br>(*Rh. mariaedominicae* Sowa & Degrange, 1987)	19
052ITVANI	Italy	Aosta Valley	45.785	6.900	hybrida	*Rh. nivata* (Eaton, 1871)	13
078FRHSLA	France	Haute-Savoie	46.098	6.592	alpestris	*Rh. landai* Sowa & Soldán, 1984<br>(*Rh. vaillanti* Sowa & Degrange, 1987)	27
088CZBOBE	Czech Republic	South Bohemia	48.671	14.615	diaphana	*Rh. beskidensis* Alba-Tercedor & Sowa, 1987	29
094PLPPIR	Poland	Lesser Poland	49.251	19.815	semicolorata	*Rh. iridina* (Kolenati, 1839)	6
110FRSAGT	France	Savoie	45.688	6.117	hybrida	*Rh. gratianopolitana* Sowa, Degrange & Sartori, 1986	16
111FRSAGT	France	Savoie	45.688	6.117	hybrida	*Rh. gratianopolitana*	17
113FRSADO	France	Savoie	45.639	6.192	semicolorata	*Rh. dorieri* Sowa, 1971<br>(*Rh. colmarsensis* Sowa, 1984)	1
117FRPYLO	France	Pyrenees	42.780	1.132	loyolaea	sp 9	25

118CZPAPT	Czech Republic	Pardubice	50.093	16.815	semicolorata	*Rh. puytoraci* Sowa & Degrange, 1987	11
163FRARTA	France	Ardèche	44.837	4.646	semicolorata	sp 4	9
173CHFRSC	Switzerland	Fribourg	46.520	6.969	semicolorata	sp 5	12
204FRPYSP	France	Pyrenees	42.780	1.132	semicolorata	sp 2	4
266FRAHDI	France	Alpes de Haute-Provence	44.330	6.587	hybrida	sp 6	21
303FRDRFO	France	Drôme	44.746	5.503	semicolorata	*Rh. fonticola* Sowa & Degrange, 1987	8
317CHVSAP	Switzerland	Valais	46.175	7.576	alpestris	*Rh. alpestris* Eaton, 1885	28
373FRHSSA	France	Haute-Savoie	45.892	6.254	diaphana	*Rh. savoiensis* Alba-Tercedor & Sowa, 1987	31
400ITVESP	Italy	Veneto	46.624	12.469	semicolorata	sp 3	7
450AUVOGE	Austria	Voralberg	47.493	9.709	semicolorata	*Rh. germanica* Eaton, 1885	2
461CHVSLO	Switzerland	Valais	46.214	7.968	loyolaea	sp 8	24
494CZKRCR	Czech Republic	Hradec Králové	50.679	15.833	hybrida	*Rh. corcontica* Sowa & Soldán, 1986	22
555CHGEPI	Switzerland	Genève	46.346	6.152	semicolorata	*Rh. picteti* Sowa, 1971	10
**576CHBEBE**	**Switzerland**	**Bern**	**47.049**	**7.634**	**diaphana**	**sp 10**	**30**
**581FRCOIN**	**France**	**Corsica**	**42.275**	**8.890**	**hybrida**	***Rh. insularis* Esben-Petersen, 1913**	
**583FRCOEA**	**France**	**Corsica**	**42.297**	**8.699**	**alpestris**	***Rh. eatoni* Esben-Petersen, 1912**	
**615ITSRNU**	**Italy**	**Sardinia**	**39.987**	**9.187**	**hybrida**	***Rh. nuragica* Belfiore, 1987**	
**625ITTODE**	**Italy**	**Tuscany**	**44.130**	**10.715**	**hybrida**	**sp 11**	
**655SKPRTT**	**Slovakia**	**Prešov**	**49.265**	**20.141**	**loyolaea**	**sp 7**	**23**
**656TUJETU**	**Tunisia**	**Jendouba**	**36.802**	**8.657**	**sowai**	***Rh. sartorii* Zrelli & Boumaiza, 2011**	
**662MTZASP**	**Montenegro**	**Žabljak**	**43.152**	**19.090**	**semicolorata**	**sp 12**	
**668ROCLSP**	**Romania**	**Cluj**	**46.796**	**22.731**	**semicolorata**	**sp 13**	
**688UKPGSE**	**United Kingdom**	**Wales**	**51.899**	**-3.114**	**semicolorata**	***Rh. semicolorata* (Curtis, 1834);** sp 1 in Vuataz *et al*. (2011)	**3**
**780FRISDE**	**France**	**Isère**	**45.209**	**6.058**	**hybrida**	***Rh. degrangei* Sowa, 1969**	**14**

Table S2. Genbank/ENA accession numbers of the five gene fragments. “HM” and “JF” codes are from Vuataz et al. [1], “LN” and “KT” codes are from the present study. The voucher name (voucher) and the species identification as labeled in Figs. 2, 3 (sp) are also provided.

**voucher**	**sp**	***cox1***	**16S**	**PEPCK**	**EF-1α**	**wg**
033FRDRDI	*diensis*	HM480867	LN868637	HM582956	LN868559	LN868598
037AUTYRO	*taurisca* (+ *rolandi*)	HM480871	LN868638	HM582960	LN868560	LN868599
041CHGRGS	*grischuna*	HM480875	LN868639	KT159745	LN868561	LN868600
042FRHSAB	*allobrogica*	HM480876	LN868640	HM582964	LN868562	LN868601
045PLPPCI	*circumtatrica*	HM480877	LN868641	KT159746	LN868563	LN868602
047FRISMA	*hybrida* (+ *austriaca*, *endenensis*, *mariaedominicae*)	HM480878	LN868642	KT159747	LN868564	LN868603
052ITVANI	*nivata*	LN868541	LN868643	KT159748	LN868565	LN868604
078FRHSLA	*landai* (+ *vaillanti*)	HM480898	LN868644	HM582987	LN868566	LN868605
088CZBOBE	*beskidensis*	LN868542	LN868645	HM582994	LN868567	LN868606
094PLPPIR	*iridina*	HM480909	LN868646	HM582999	LN868568	LN868607
110FRSAGT	*gratianopolitana*	HM480921	LN868647	KT159749	LN868569	LN868608
111FRSAGT	*gratianopolitana*	HM480922	LN868648	KT159750	LN868570	LN868609
113FRSADO	*dorieri* (+ *colmarsensis*)	HM480924	LN868649	HM583011	LN868571	LN868610
117FRPYLO	sp 9	HM480928	LN868650	HM583014	LN868572	LN868611
118CZPAPT	*puytoraci*	HM480929	LN868651	HM583015	LN868573	LN868612
163FRARTA	sp 4	HM480960	LN868652	HM583045	LN868574	LN868613
173CHFRSC	sp 5	HM480967	LN868653	KT159751	LN868575	LN868614
204FRPYSP	sp 2	HM480986	LN868654	HM583078	LN868576	LN868615
266FRAHDI	sp 6	HM481018	LN868655	HM583126	LN868577	LN868616
303FRDRFO	*fonticola*	HM481037	LN868656	HM583150	LN868578	LN868617
317CHVSAP	*alpestris*	LN868543	LN868657	KT159752	LN868579	LN868618
373FRHSSA	*savoiensis*	LN868544	LN868658	HM583189	LN868580	LN868619
400ITVESP	sp 3	HM481077	LN868659	KT159753	LN868581	LN868620
450AUVOGE	*germanica*	LN868545	LN868660	HM583241	LN868582	LN868621
461CHVSLO	sp 8	LN868546	LN868661	KT159754	LN868583	LN868622
494CZKRCR	*corcontica*	HM481129	LN868662	HM583279	LN868584	LN868623

555CHGEPI	*picteti*	LN868547	LN868663	HM583329	LN868585	LN868624
576CHBEBE	sp 10	LN868548	LN868664	KT159756	LN868586	LN868625
581FRCOIN	*insularis*	LN868549	LN868665	KT159757	LN868587	LN868626
583FRCOEA	*eatoni*	LN868550	LN868666	KT159758	LN868588	LN868627
615ITSRNU	*nuragica*	LN868551	LN868667	KT159759	LN868589	LN868628
625ITTODE	sp 11	LN868552	LN868668	KT159760	LN868590	LN868629
655SKPRTT	sp 7	LN868553	LN868669	KT159761	LN868591	LN868630
656TUJETU	*sartorii*	LN868554	LN868670	KT159762	LN868592	LN868631
662MTZASP	sp 12	LN868555	LN868671	KT159763	LN868593	LN868632
668ROCLSP	sp 13	LN868556	LN868672	KT159764	LN868594	LN868633
688UKPGSE	*semicolorata*	LN868557	LN868673	KT159765	LN868595	LN868634
780FRISDE	*degrangei*	LN868558	LN868674	KT159755	LN868596	LN868635
m185 (outgroup)	*Cinygmula* sp.	JF423908	LN868675	JF423909	LN868597	LN868636

Table S3. Mean (mean), minimum (low) and maximum (high) altitude records for each GMYC species (sp) of the hybrida and alpestris species groups (sp group) used in the ancestral state reconstructions. The number of sampling sites (n) is also provided for each GMYC species. All altitude records are from the authors' personal database (unpublished).

**sp**	**sp group**	**mean**	**low**	**high**	**n**
grischuna	hybrida	1509	1154	1773	10
nivata	hybrida	1780	1581	2170	6
insularis	hybrida	816	625	1100	4
nuragica	hybrida	560	560	560	1
degrangei	hybrida	1224	231	2184	22
corcontica	hybrida	1147	460	1510	17
gratianopolitana	hybrida	718	400	1230	17
gratianopolitana	hybrida	893	631	1180	3
circumtatrica	hybrida	1030	950	1158	3
hybrida	hybrida	972	355	1850	46
sp 11	hybrida	850	850	850	1
diensis	hybrida	814	759	868	2
sp 6	hybrida	1685	1657	1713	2
alpestris	alpestris	1246	725	1608	12
landai	alpestris	555	12	1171	23
eatoni	alpestris	333	40	625	2
allobrogica	alpestris	473	12	653	11

Table S4. Results of birth-death likelihood (BDL) analysis of the model-completed *Rhithrogena* data set (74 taxa), based on 1000 completed phylogenies generated with CorSiM [2, 3]. *r* = diversification rate (species per My), *r*1 = diversification rate before st; *r*2 = diversification rate after st; st = time of rate shift (in Mya); *a* = extinction fraction; *x* = rate change parameter; *k* = carrying capacity. The preferred model (Yule-2-rates) is indicated in bold.

Models	Yule	**Yule-2-rates**			Birth-death		DDL		DDX	
Mean AIC	-112.18	**-113.21**			-111.84		-111.46		-110.16	
SD	6.73	**7.92**			7.78		7.57		6.69	
%	33.00	**62.00**			6.00		0.00		0.00	
Parameters	*r*	***r*1**	***r*2**	**st**	*r*	*a*	*r*	*x*	*r*	*k*
Mean	0.20	**0.21**	**0.19**	**0.22**	0.16	0.37	0.13	-0.15	0.21	1192795
SD	0.01	**0.01**	**0.22**	**0.13**	0.02	0.15	0.02	0.07	0.01	358701.07

References

1. Vuataz L, Sartori M, Wagner A, Monaghan MT. Toward a DNA taxonomy of Alpine Rhithrogena (Ephemeroptera: Heptageniidae) using a mixed Yule-coalescent analysis of mitochondrial and nuclear DNA. PLoS One. 2011;6(5):e19728.
2. Stadler T. Simulating trees with a fixed number of extant species. Syst Biol. 2011;60(5):676–84.
3. Cusimano N, Stadler T, Renner SS. A new method for handling missing species in diversification analysis applicable to randomly or nonrandomly sampled phylogenies. Syst Biol. 2012;61(5):785–92.
