# Supplementary figures and images for: Molecular phylogeny and timing of diversification in Alpine Rhithrogena (Ephemeroptera: Heptageniidae)
Source: BMC Evol Biol. 2016 Sep 21;16:194. doi: 10.1186/s12862-016-0758-1 (PMC5031269; doi:10.1186/s12862-016-0758-1)

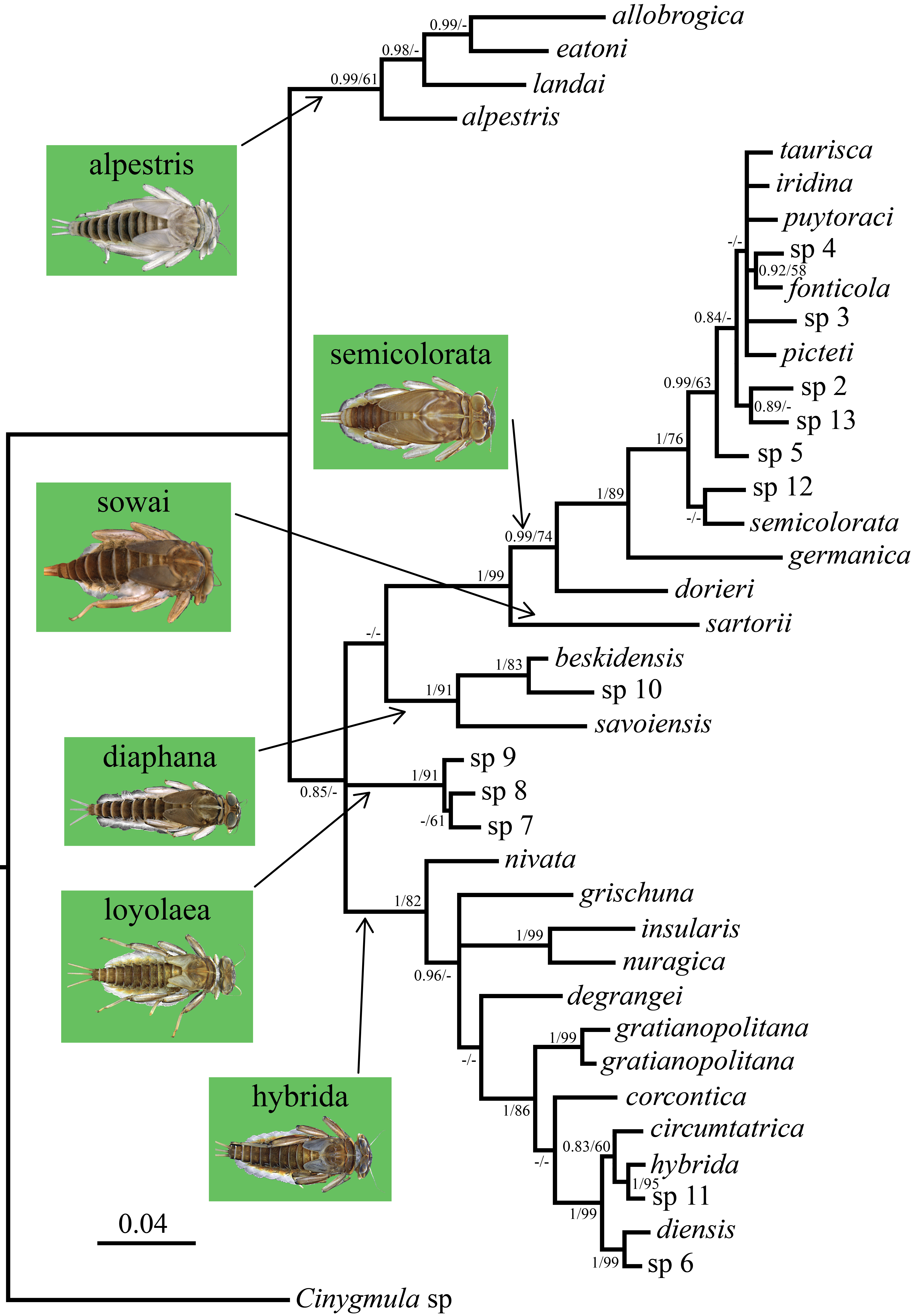

Supplement: Additional file 2: Figure S1. — Bayesian majority-rule consensus tree reconstructed from the concatenated mitochondrial (cox1 + 16S; 1169 bp) data set. Species groups are indicated with green shading, including a photograph of a species group member. For each node, Bayesian posterior probability (PP) and maximum likelihood bootstrap support (BS) values are given (PP/BS) if PP > 0.8 and BS > 60. (TIF 7398 kb) [file 12862_2016_758_MOESM2_ESM.tif]

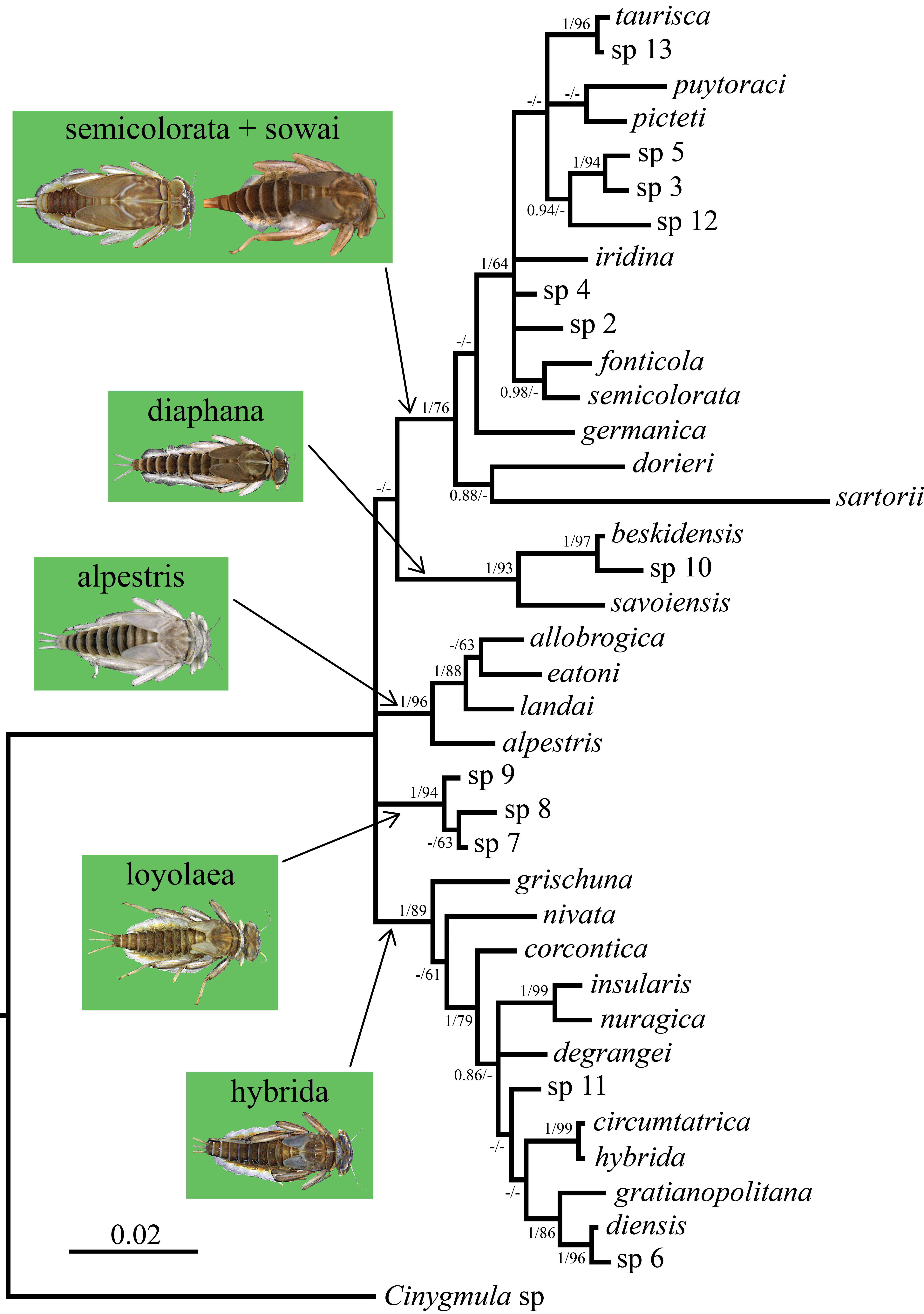

Supplement: Additional file 3: Figure S2. — Bayesian majority-rule consensus tree reconstructed from the concatenated nuclear (PEPCK + EF-1α + wg; 1147 bp) data set. Species groups and node support are indicated as in Additional file 2: Figure S1. (TIF 7255 kb) [file 12862_2016_758_MOESM3_ESM.tif]

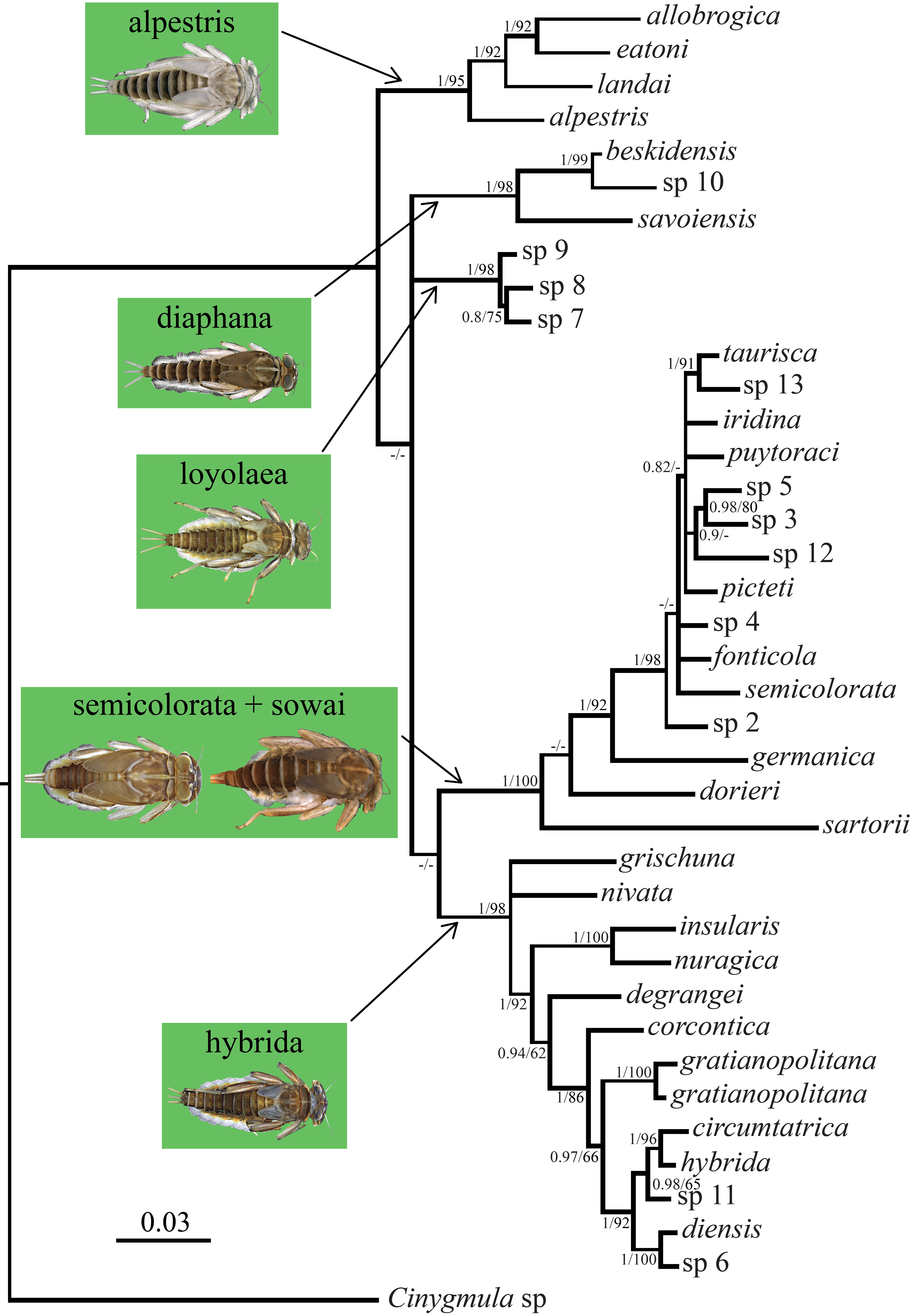

Supplement: Additional file 4: Figure S3. — Bayesian majority-rule consensus tree of the mitochondrial + nuclear concatenated (2316 bp) data set. Species groups and node support are indicated as in Additional file 2: Figure S1. (TIF 7566 kb) [file 12862_2016_758_MOESM4_ESM.tif]

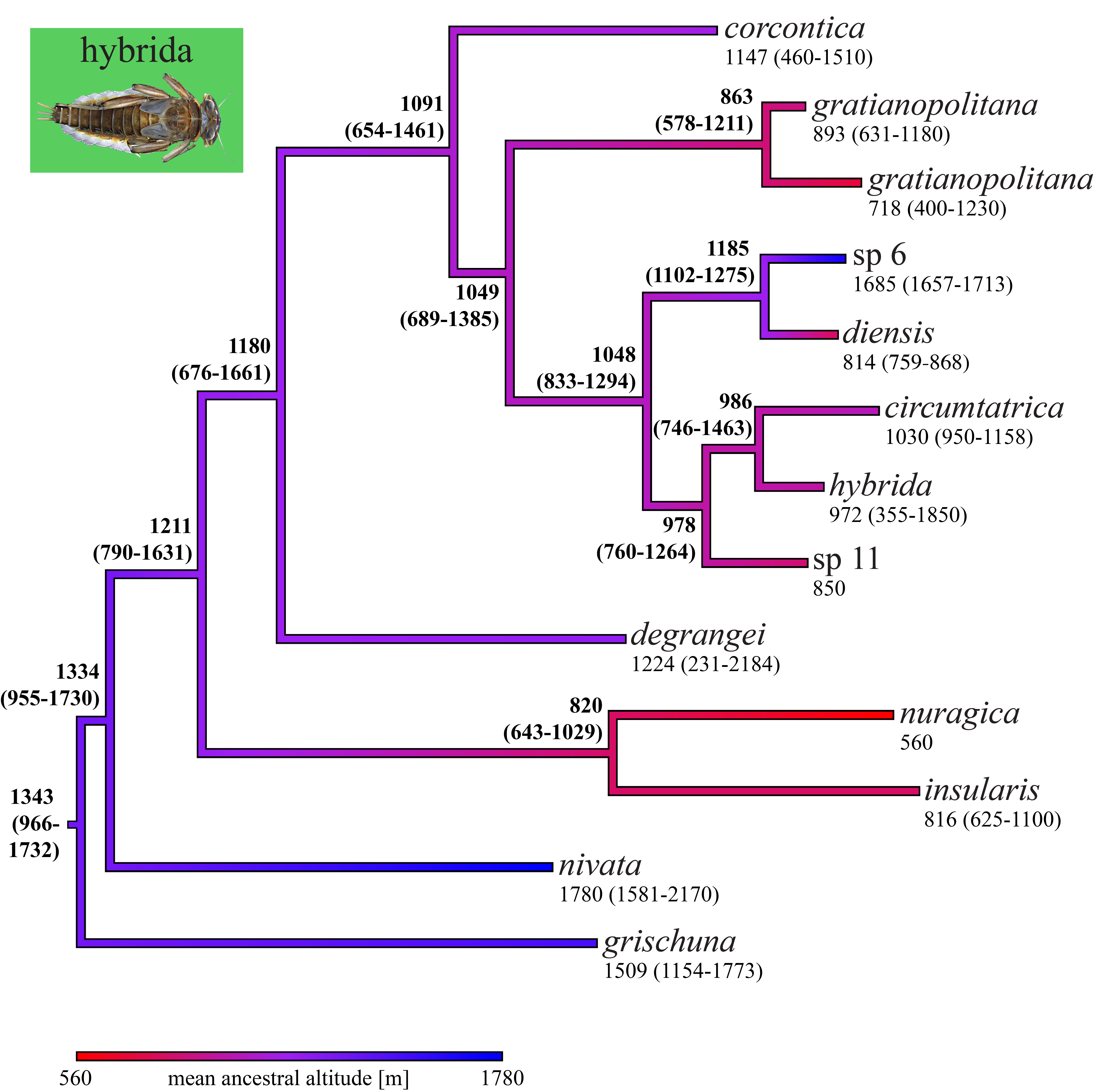

Supplement: Additional file 5: Figure S4. — ML tree of the hybrida species group extracted from the concatenated (five-gene supermatrix) ML phylogram, with ancestral altitudes reconstructed using the REML method. Mean ancestral altitudes (with minimum and maximum ancestral altitudes between brackets) are indicated for each node. Mean altitudes (with altitude ranges between brackets) extracted from authors’ own records (see Additional file 1: Table S3), and used as raw data for the ancestral altitude reconstruction, are given below each terminal GMYC species. The color gradient symbolizing the mean altitude gradient along the tree branches was obtained using the contMap function of the phytools package [129] for R. (TIF 3301 kb) [file 12862_2016_758_MOESM5_ESM.tif]

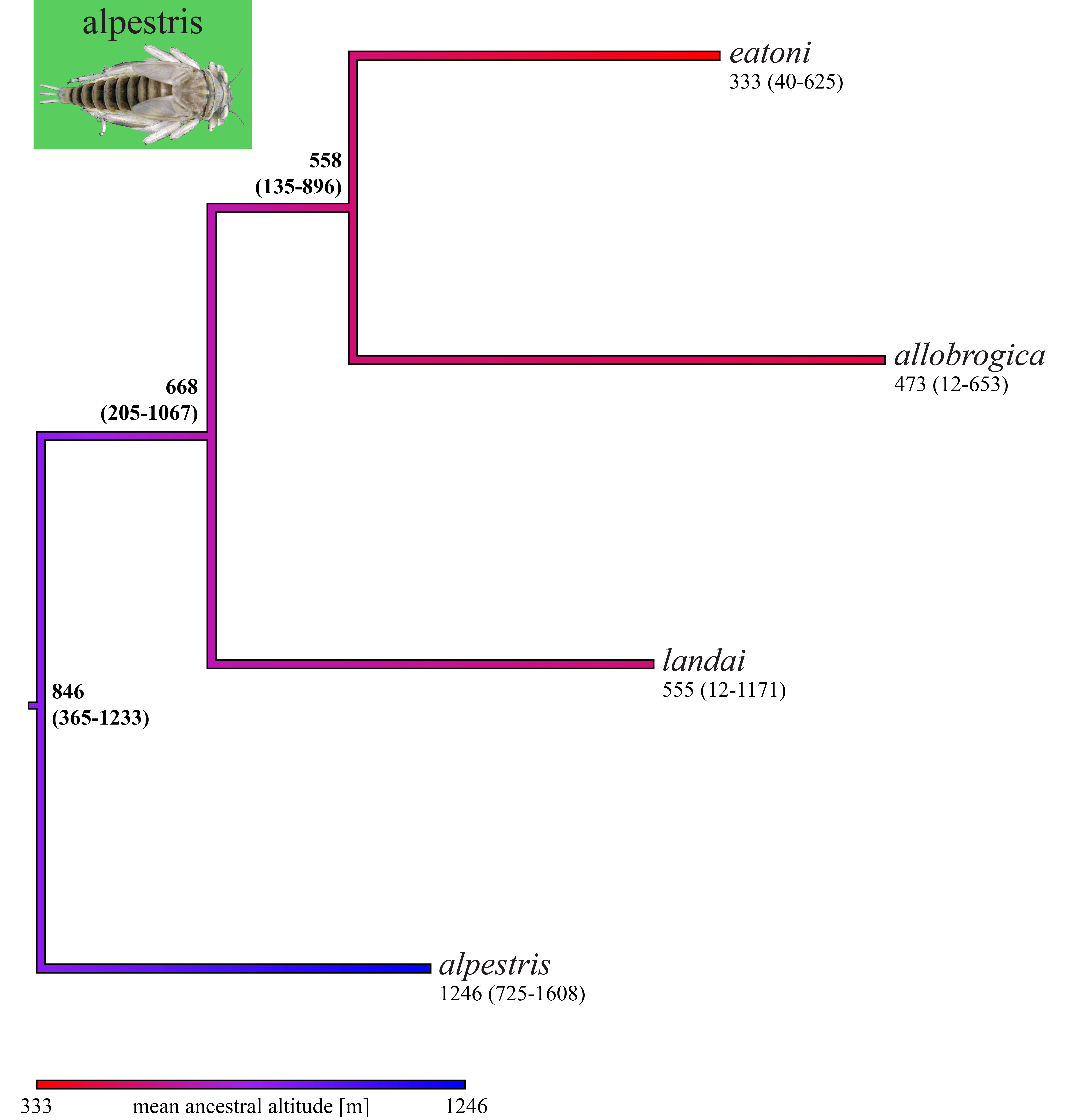

Supplement: Additional file 6: Figure S5. — ML tree of the alpestris species group extracted from the concatenated (five-gene supermatrix) ML phylogram, with ancestral altitudes reconstructed using the REML method. The altitudes and the color gradient are indicated as in Additional file 5: Figure S4. (TIF 2765 kb) [file 12862_2016_758_MOESM6_ESM.tif]
